# Field Evaluation of the Interferon Gamma Assay for Diagnosis of Tuberculosis in Water Buffalo (*Bubalus bubalis*) Comparing Four Interpretative Criteria

**DOI:** 10.3389/fvets.2020.563792

**Published:** 2020-12-01

**Authors:** Alessandra Martucciello, Nicoletta Vitale, Piera Mazzone, Alessandro Dondo, Ivonne Archetti, Laura Chiavacci, Anna Cerrone, Fabrizio Gamberale, Lorena Schiavo, Maria Lodovica Pacciarini, Maria Beatrice Boniotti, Esterina De Carlo

**Affiliations:** ^1^National Reference Centre for Hygiene and Technologies of Water Buffalo Farming and Productions, Istituto Zooprofilattico Sperimentale del Mezzogiorno, Salerno, Italy; ^2^Istituto Zooprofilattico Sperimentale del Piemonte, Liguria e Valle d'Aosta, Turin, Italy; ^3^Istituto Zooprofilattico Sperimentale dell'Umbria e delle Marche “Togo Rosati”, Perugia, Italy; ^4^National Reference Centre for Bovine Tuberculosis, Istituto Zooprofilattico Sperimentale della Lombardia e dell'Emilia Romagna, Brescia, Italy; ^5^Istituto Zooprofilattico Sperimentale di Lazio e Toscana, Rome, Italy

**Keywords:** water buffalo, tuberculosis, diagnosis, interferon-gamma (IFN-γ) test, *Mycobacterium bovis*, *Mycobacterium avium* subsp. *paratuberculosis*, ESAT6 CFP10

## Abstract

Bovine tuberculosis (bTB) is a worldwide zoonosis that affects many species of domestic and wild animals. *Mycobaterium bovis* is the main cause of infection in water buffalo (*Bubalus bubalis*) and bovines and is of great concern for human health and for buffalo producers in Italy. The bTB eradication programme is based on slaughterhouse surveillance and intradermal skin tests. Other *in vivo* diagnostic methods such as the interferon-gamma (IFN-γ) assay have been developed and are widely used in cattle to accelerate the elimination of bTB positive animals. The present study is the first to assess the use and performance of IFN-γ assays, which is used as an ancillary test for bTB diagnosis in water buffalo, and presents the results of a field-evaluation of the assay from 2012 to 2019 during the buffalo bTB eradication programme in Italy. The study involved 489 buffaloes with a positive result to the single intradermal tuberculin test (SITT). The IFN-γ assays and single intradermal comparative tuberculin test were used as confirmation tests. Then, a total of 458 buffaloes, reared on officially tuberculosis-free (OTF) herds, that were confirmed bTB-free for at least the last 6 years were subjected to IFN-γ testing. Furthermore, to evaluate the IFN-γ test in an OTF herd with Paratuberculosis (PTB) infection, 103 buffaloes were subjected to SITT and IFN-γ test simultaneously. Four interpretative criteria were used, and the IFN-γ test showed high levels of accuracy, with sensitivity levels between 75.3% (CI 95% 71.2–79.0%) and 98.4% (CI 95% 96.7–99.4%) and specificity levels between 94.3% (CI 95% 91.2–96.50%) and 98.5% (CI 95% 96.9–99.4%), depending on the criterion used. Finally, in the OTF herd with PTB infection, in buffalo, the IFN-γ test displayed high specificity values according to all 4 interpretative criteria, with specificity levels between 96.7% (CI 95% 88.4–99.5%) and 100% (CI 95% 96.2–100%), while SITT specificity proved unsatisfactory, with a level of 45.3% (CI 95% 35.0–55.7%). Our results showed that the IFN-γ test in the buffalo species could reach high Sensitivity and Specificity values, and that the level of Sensitivity and Specificity could be chosen based on the interpretative criterion and the antigens used depending on the health status of the herd and the epidemiological context of the territory. The IFN-γ test and the use of different interpretative criteria proved to be useful to implement bTB diagnostic strategies in buffalo herds, with the possibility of a flexible use of the assay.

## Introduction

Tuberculosis (TB) is a zoonosis of global importance, causing major economic losses and trade restrictions. In the year 2018, 10 million people contracted TB worldwide, of whom 1.5 million died (1). This data includes human TB caused by both *Mycobacterium tuberculosis* and *Mycobacterium bovis*; therefore, one of the objectives of the WHO is to improve the surveillance and reporting of bovine TB (bTB) in livestock and wildlife and to augment the capacity of the animal health sector to reduce the prevalence of bTB.

The first description of bTB in water buffalo (*Bubalus bubalis*) was recorded in Egypt in 1986 (2). Since then several studies reported the increasing prevalence of bTB in buffalo in many parts of the world mainly due to *M. bovis* (3–6).

About 73% of the Italian buffalo stock is bred in the Campania region in the South of Italy, according to the Italian National Livestock Database (7), where this species is of great economic importance. Indeed, buffalo rearing constitutes an important zootechnical and economic resource in a large area of central-southern Italy, where it has replaced the bovine species in the production of many dairy products, mainly the typical “buffalo mozzarella”.

The prevalence and incidence rates of bTB in the Campania buffalo population were 7.30 and 3.50%, respectively, in 2018 and 13.80 and 8.9% in 2019. It should be noted that about 6,500 animals were slaughtered in 2018 and 4,600 in 2019 as a result of “test and cull” strategy adopted during bTB outbreaks. The cost to the Campania region for compensating buffalo farmers exceeded €20 million in the 2019 (data from Campania region report). It would therefore be extremely advantageous to have an accurate diagnostic strategy that could rapidly reveal bTB outbreaks during the early stage of *M. bovis* infection, in order to acquire the health status of Officially Tuberculosis-Free (OTF) territory. Improve the diagnosis of bTB is a real challenge because this disease is still causing serious economic and genetic losses as a result of the slaughter of infected animals and the depreciation of milk, which is subject to obligatory heat treatment on *M. bovis* infected farms, in a territory whose economy is closely linked to buffalo dairy products.

The oldest test for the ante mortem diagnosis of TB is the single intradermal tuberculin test (SITT), recognized by the World Organization of Animal Health and the European Commission as the screening test, used in swamp buffalo (8, 9) and in water buffalo (6). However, in buffalo species, SITT has been reported with low sensitivity (Se) and specificity (Sp) either in *Syncerus caffer* or in *B. bubalis* (9, 10).

In buffalo species, it was suggested that malnutrition leads to ineffective immune response and it could yield a false negative result, while sensitization with non-tuberculosis mycobacteria (NTM), such as *M. terrae, M. nonchromogenicum, M. vaccae, Mycobacterium avium* subsp. *avium*, and *M. avium* subsp. *paratuberculosis* (MAP), might reduce its Sp (11, 12). Moreover, the execution and interpretation of the intradermal test can be affected by the varying thickness of the skin in buffalo and cattle, by the black color of the buffalo skin, and the harder tissue structure. In fact, in the middle third of the cervical region, skin thickness ranges between 15 and 30 mm in buffalo vs. 5–8 mm in cattle (13).

The single intradermal comparative tuberculin test (SICTT) is used primarily as an ancillary test for positive or inconclusive reactors in the SITT [(14) 64/432/EEC]. SICTT provides a better discrimination between animals infected with *M. bovis* and those infected with *M. avium* complex or environmental mycobacteria (15–17), increasing the Sp but with a still low Se (15, 18, 19). In Brazil, it has been reported that SICTT has 71.43% Se and 82.61% Sp in water buffalo (10).

Currently, *in vitro* indirect screening tests like interferon gamma-release assays (IGRAs) are also available to detect mycobacterial infections. In human medicine, QuantiFERON®-TB Gold In-Tube test (QFT-GIT) and the T-SPOT-TB test (ELISPOT) (20) are used routinely. In veterinary medicine, in addition to SIT and SICT tests, the interferon-gamma (IFN-γ) assay, developed in Australia in the early 1990s (21) has been widely used in bovine species for the diagnosis of bTB (18, 22–27).

The World Organization of Animal Health's Terrestrial Manual included the IFN-γ assay since 1996, and the European Union adopted it as an ancillary test to the SITT since 2002 (28) to improve the detection of bTB-infected animals in a herd or in a region (29). It is believed that the IFN-γ test has the ability to provide an early detection of bTB compared to intradermal skin tests (23, 30), in fact, in several countries it is used for serial or parallel testing together with SIT or SICT tests (31, 32).

Several studies (15, 30, 33) have reported the utility of the IFN-γ test in bTB diagnosis in cattle, with a Se median value of 87.6% (range 73.0–100%) and a Sp median value of 96.6% (range: 85.0–99.6%).

However, in the literature, regarding IFN-γ test in buffalo species, scarce data are available and mainly associated with African buffaloes (*S. caffer*), (34–41).

Even though *S. caffer* and *B. bubalis* are distinguished by taxonomic classification (42) with different phenotypic traits, they belong to the same family *Bovidae* and sub-family *Bovinae*, as *Bos taurus* and *Bos indicus*. Hence, a different behavior of the cell-mediated immune response (CMI) to *M. bovis* infection is not expect.

The intradermal skin tests and IFN-γ test measure the cell-mediated immune response (CMI) to *M. bovis* infection (43). The IFN-γ test detects the cytokine produced by the T lymphocytes of infected subjects in response to stimulation with tuberculin antigens (15). The tuberculins used in both SITT and SICTT, as in the IFN-γ test, are the purified protein derivatives (PPDs) extracted from mycobacteria cultures in liquid synthetic medium. Bovine PPD (PPDB) is obtained from *M. bovis* AN5, while avian PPD (PPDA) is extracted from *M. avium* D4ER (44). In the IFN-γ test, stimulation is performed with both tuberculin antigens, PPDB and PPDA, to compare the different immune responses likewise is done in SICTT (25, 29, 45, 46).

Despite the standardization of production of PPD tuberculins and their regulation by EU (14), the estimated potency can vary between different manufacturers (15, 18, 47, 48). This could affect the performance of the IFN-γ test as described earlier (49) especially when more than one couple of PPDs were used. For this reason, we wanted to verify whether, in buffalo, the use of two couples of PPDs (Lelystad and Italian PPDB and PPDA) could give different results in the IFN-γ test and eventually improve the accuracy in different epidemiological scenarios.

The EFSA Scientific Opinion on the IFN-γ test (29) states “In infected herds (containing reactors already disclosed by tuberculin tests) the test can be applied in different ways, depending on the suspected level of infection in the herd.” Therefore, to optimize the utilization of the IFN-γ test in infected herds or in OTF herds, an optimal cut-off value or an optimal interpretative criterion could be obtained with the analysis of receiver operating characteristic (ROC) curve (29, 32, 50). In this regard, several modifications to the original IFN-γ test protocol have been introduced in cattle (45, 46, 51, 52) with different cut-offs and thresholds (53) and different PPDs (49). This was done to optimize the performance and for the application of the assay in different contexts of bTB prevalence (29).

Therefore, even in buffalo, it has been useful to evaluate different interpretative criteria and cut-off values of the IFN-γ test, to adapt the assay to this animal species and in different epidemiological scenarios.

In addition, in order to improve the Sp of the IFN-γ test, specific antigens, such as 6 kDa early secretory antigenic target (ESAT-6) and 10 kDa culture filtrate protein (CFP-10) have been used during whole blood stimulation (54–57). Both EAST6 and CFP10 antigens (58, 59) are expressed in *M. bovis* but absent from NTM and *M. bovis* Bacillus Calmette Guerin. These antigens have been proposed as relevant in differentiating infected and vaccinated animal test candidates (60, 61) and used as alternative or additional antigens to the PPDs for blood stimulation in the IFN-γ assay in cattle (16, 30, 62, 63) and in African buffaloes (36).

To the best of our knowledge, there is no published literature evaluating the IFN-γ test in water buffaloes in which ESAT6/CFP10 were also used in addition to PPDs for blood stimulation.

The aim of this study was to evaluate the performance of the IFN-γ test in healthy buffaloes and naturally *M. bovis*-infected buffaloes. Therefore, we developed an IFN-γ test with combination of PPDs, a mixture of ESAT-6 and CFP-10 and four different interpretative criteria.

The final goal was to verify the use of the IFN-γ test as an ancillary test to implement bTB diagnostic strategies in buffalo herds.

## Materials and Methods

### Animal Population Characteristics and Ethics Statement

The test results used for the present investigation were collected within the context of the officially ordered tuberculosis-surveillance program in accordance with Italian National [(28, 64, 65), (66): Order 9 August 2012] and Regional regulations (DD Campania n. 236/2016^1^; DD Campania n. 226/2016^2^).

Animal owners were informed of the tests carried out and all the samples were collected during the mandatory health investigations.

A total of 1,050 Mediterranean water buffaloes (*B. bubalis*) were selected in Italy from 2012 to June 2019. We evaluated the use of different PPDs and recombinant antigens and assessed the accuracy of 4 different interpretative criteria of the IFN-γ test under field conditions in herds with bTB outbreaks in OTF herds and 1 OTF herd with Paratuberculosis (PTB) infection.

The National and regional buffalo tuberculosis-surveillance program provided by Italian Ministry of Health and Campania Region [(64, 65); DD Campania n. 236/2016; DD Campania n. 226/2016] required the use of SITT as a screening test and tested positive result in OTF herds, has to be confirmed and retested. Therefore, following a positive SITT finding, the OTF herd became the “herd with suspected outbreak of TB” and sanitary restrictions were mandated, waiting for further confirmation tests such as SICTT and the IFN-γ test, after at least 42 days. The IFN-γ test was used as part of an experimental protocol authorized by the Italian Ministry of Health and the Campania region. In accordance with national and regional legislation, animals found to be positive on any one of the confirmation test were slaughtered.

A confirmed bTB outbreak was defined as a farm with positive SITT and SICTT and/or a positive IFN-γ test confirmed by isolation of *M. bovis* in at least one animal.

### Accuracy of Four Interpretative Criteria of the IFN-γ Test

The diagnostic Se of the IFN-γ test performed using four different interpretative criteria was calculated in a subpopulation of *M. bovis*-infected buffaloes, which were SITT and post-mortem positive. Therefore, the Se values reported in this paper refer to this subpopulation.

Hence, to assess the IFN-γ test interpretative criteria Se, we used a data set comprised of 489 bTB infected buffaloes, from 71 herds (range 1–35 animals) of confirmed bTB outbreaks in the Campania region.

Complying with the National and regional regulations mentioned above, provided by Campania Region from 2012 to June 2019, we could only include those animals that, during the activities of the regional buffalo tuberculosis-surveillance program, resulted positive to the SITT screening test in OTF herds. As stated previously, for SITT positive animals, a second access into the OFT herd, at least 42 days after the SITT, was required; official veterinarians carried out SICTT and blood sampling for IFN-γ test at the same time. Animals were deemed positive if they react to at least one of the confirmatory tests (SICTT and/or IFN-γ parallel testing). Positive animals were slaughtered in accordance with national and regional legislation, and the organs were submitted to laboratory examinations at Istituto Zooprofilattico Sperimentale del Mezzogiorno.

The performance of the IFN-γ test, particularly the Se, is usually evaluated by verifying that the animals that tested positive for IFN-γ are also positive for the isolation of *M. bovis*. In fact, culture isolation is considered the gold standard for the confirmation of *M. bovis* infection status.

However, since the Se of culture examination for *M. bovis* is low (67, 68) we decided that, in the case of infected herds, an animal was considered positive if bTB lesions had been found at the slaughterhouse and/or proved to be positive on the culture test and/or PCR.

Sp of the IFN-γ test was evaluated in a population of 458 buffaloes from 4 OTF herds during the annual SITT screening test performed in the last 6 years. The farms were located in 4 Italian regions: two farms in OTF territories in northern Italy (Piedmont, Lombardy), one in central Italy (Lazio) and one in the southern of Italy (Campania). All the herds were negative for PTB on serological testing.

A negative animal was defined as a buffalo from an OTF herd and tested negative to the SITT during the last 6 years.

### Assessment of the Performance of SITT and IFN-γ on a Tuberculosis Officially Free Farm With MAP Infection

Sp of the IFN-γ test was also evaluated in a data-set of 103 buffaloes from 1 OTF herd, for the last 10 years, with PTB infection.

As NTM can interfere with bTB diagnosis (16, 41), we wanted to evaluate the performance of SITT and IFN-γ in the presence of NTM, in particular MAP, that could be present in buffalo herds (41, 69–75).

The 103 buffaloes were simultaneously tested with SITT and IFN-γ. Circulation of MAP had been confirmed by serological tests during the previous 2 years. On this farm, no buffaloes with suspect lesions of bTB had been detected at the slaughterhouse in the previous 10 years; moreover, no epidemiological link with infected farms had been established during the previous 6 years. In addition, a thorough epidemiological investigation was conducted to exclude the presence of *M. bovis* in this farm and in the neighboring farms.

### Diagnostic Methods

#### The Intradermal Tuberculin Tests

The SITT was performed by the official/national veterinary services of the territory, in accordance with EU regulations and Italian legislation: (64, 65)—(28), O. M. 9 August 2012—and subsequent amendments. A skin fold was measured with calipers before and 72 h after the inoculation of 0.1 ml (30,000 I.U./ml) of PPDB (Istituto Zooprofilattico Sperimentale dell'Umbria e delle Marche, Italy).

The intradermal injection was performed by means of hypodermic needles mounted on Inj-Light syringes, at the border of the anterior and middle third of the neck, over the left shoulder of the animal, near the acromion spina scapulae (76). Results were expressed in millimeters as the difference between the two measurements, i.e., before and 72 h after the inoculation of tuberculin. The reaction was considered positive if skin thickness increased by ≥ 4 mm, inconclusive if >2 and <4 mm, and negative if ≤ 2 mm.

SICTT was also performed, but only on animals with a positive SIT screening test, from suspected-infected or infected herds.

The avian tuberculin (PPDA Istituto Zooprofilattico Sperimentale dell'Umbria e delle Marche, Italy) was inoculated into the right shoulder. In accordance with National and Regional regulations, the reaction was considered positive if the difference between the PPDB and PPDA measurements was ≥ 4 mm, inconclusive if <4 and >1 mm, and negative if ≤ 1 [(28, 64, 65), O. M. 9 August 2012—and subsequent amendments).

#### IFN-γ Test

Heparinized blood samples were collected from each animal before the inoculation of the tuberculin and transported to the laboratory at room temperature within 8 h of collection.

Blood samples were dispensed under a laminar-flow hood in 1 ml aliquots on cell-culture plates and stimulated with two different couples of Avian and Bovine PPDs, provided by Thermo-Fisher Scientific (Lelystadt PPDs: final concentration 10 μg/ml) and by Istituto Zooprofilattico dell'Umbria e delle Marche, Italy, produced and purified as described by Corneli et al. (44) (Italian PPDs: final concentration 10 μg/ml PPDB and 5 μg/ml PPDA).

In addition, the ESAT6/CFP10 protein cocktail, produced and purified as described by Fontana et al. (77) (final concentration of each protein 4 μg/ml), was also used to stimulate blood samples.

Phosphate buffer saline (PBS), used as Nil Control Antigen (NIL), that represented the IFN-γ basal value in the single animal. A control of lymphocyte viability (pokeweed mitogen: PWM, final concentration 1 μg/ml) was also included in order to control the ability of blood cells to produce IFN-γ. In particular, PWM detects the possible presence of lymphocyte-inhibiting substances due to the illegal use of immunodepressive drugs and reveals the reduction in the immune response against various physiological or pathological conditions (57, 78).

The culture plates were incubated for 16 to 24 h at 37°C in a humidified atmosphere.

After incubation, the culture plates were centrifuged at 500 × g for 10 min at room temperature (22 ± 5°C); the culture supernatant, i.e., the plasma of each sample, was collected.

The levels of IFN-γ in culture supernatants, were measured by means of a sandwich enzyme linked immunosorbent assay (ELISA) according to the instruction of manufacturer (Bovigam, Thermo-Fisher Scientific, Schlieren, Switzerland).

The absorbance of each well was read with a 450 nm filter, and the absorbance values, expressed as optical density (OD) units, were used to calculate the results.

The quality control of ELISA assay was applied according to the instruction provided by manufacturing company which requires a range of acceptability of OD values <0.130 for negative controls (NC) and > 0.700 for positive controls (PC). Results were excluded when the OD value for the PWM-treated sample was < 0.5 OD (45).

Four different interpretative criteria (Table 1) were used, in particular in the first, second, and third criterion a comparison between PPDB and PPDA was performed, applying different cut-offs to interpret the results. In the fourth criterion, the comparison was between recombinant antigens and the basal value (PBS).

**Table 1:** IFN-γ test interpretative criteria adopted in the study.

**Criteria**	**INTERPRETATIVE CRITERIA**	
Criterion 1	PPDB-PBS ≥ 0.1 and PPDB-PPDA ≥ 0.1 = **POSITIVE**	
	PPDB-PBS < 0.1 = **NEGATIVE**	
	PPDB-PPDA < 0.1 = **NEGATIVE**	
Criterion 2	PPDB ≥ 2*PBS and (PPDB-PPDA) ≥ 0.050 = **POSITIVE**	
	PPDB ≤ 2*PBS = **NEGATIVE**	
	PPDB ≤ PPDA = **NEGATIVE**	
	PPDB ≥ 2*PBS and 0.001 ≤ (PPDB-PPDA) ≤ 0.049 = **INCONCLUSIVE**	
Criterion 3	If the basal value exceeds 0.150 OD before stimulation, the sample is considered UNSUITABLE	
	First level	PPDB and PPDA < 2*PBS = **NEGATIVE**
		PPDB ≥ 2*PBS = **BOVIS**
		PPDA ≥ 2*PBS = **AVIUM**
	If PPDB and PPDA > 2*PBS then do PPDB/PPDA	If PPDB/PPDA ≤ 0.9 = **AVIUM**
		PPDB/PPDA ≥ 1.1 = **BOVIS**
		0.9 < PPDB/PPDA <1.1 = **INCONCLUSIVE (IN)**
	Second level	If Lely PPDs = Bovis and It PPDs = Bovis then **POSITIVE**
		If Lely PPDs = Negative and It PPDs = Negative then **NEGATIVE**
		If Lely PPDs = Avium/Neg and It PPDs = Avium/Neg then **NEGATIVE**
		If Lely PPDs = IN/A/Neg and It PPDs = Bovis then **Not Discriminant (ND)**
		If Lely PPDs = Bovis and It PPDs = IN/A/Neg then **Not Discriminant (ND)**
Criterion 4	ESAT6/CFP10-PBS ≥ 0.1 = **POSITIVE**	
	ESAT6/CFP10-PBS < 0.1 = **NEGATIVE**	

The bold values are the results of IFN-γ assays according to 4 criteria

*PPD, Purified Protein Derivative; PPDB, Bovine PPD; PPDA, Avian PPD; Lely, Lelystad PPDs; and It, Italian PPDs*.

##### Criterion 1

This criterion was the interpretation suggested by the manufacturer (Bovigam, Thermo-Fisher Scientific, Schlieren, Switzerland). It considers only PPDs supplied by Lelystad (Bovigam, Thermo-Fisher Scientific, Schlieren, Switzerland) and to define the positive sample the recommended cut-off had a net difference of PPDB–PPDA ≥ 0.1 OD if PPDB—PBS ≥ 0.1 OD.

##### Criterion 2

This criterion considers only PPDs supplied by Lelystad, and to define the positive sample, the recommended cut-off was the difference of PPDB–PPDA ≥ 0.05 OD if PPDB ≥ 2^*^PBS OD. Samples with value between 0.001 OD ≤ (PPDB-PPDA) ≤ 0.049 OD were considered inconclusive (IN).

This was the interpretative criterion used by the Italian National Reference Center for bTB, for the diagnosis of bTB in cattle infected herds alongside with SITT (29, 79, 80); the thresholds used in this criterion have also been evaluated in cattle by other authors (45, 50, 52, 81, 82).

##### Criterion 3

This criterion uses two couples of PPDs (Italian and Lelystad) and considers the IFN-γ tests as two separate tests, performed simultaneously.

To define the positive sample the recommended cut-off had a net ratio of PPDB/PPDA ≥ 1.1 OD if PPDB and PPDA ≥ 2^*^PBS OD. When ratio value was between 0.9 OD < (PPDB/PPDA) < 1.1 OD, samples were considered inconclusive.

This criterion used the ratio value obtained with stimulation of blood samples with two couples of PPDs (Lelystad and Italian). When the results of PPDs Lelystad and PPDs Italian disagree, the test was considered inconclusive and was labeled as “not discriminant” (ND). Animals with an ND result must be re-tested later after at least 42 days from the time of intradermal skin tests.

Similar to other authors (16, 32), a maximum threshold of the basal value (PBS ≤ 0.150 OD) has been introduced in this criterion as an additional quality control. Therefore, animals with high basal values due to pre-existing pathologies were not considered. The value of 0.150 OD was obtained by considering the mean + 7 times the standard deviation of the baseline value of 200 animals belonging to different types of rearing practices (83).

This interpretative criterion was validated at Istituto Zooprofilattico Sperimentale del Piemonte, Liguria e Valle d'Aosta laboratory and was used to eradicate bTB in Piedmont in the years from 2004 to 2016 when the region acquired the European OTF status, according to (84), and is currently used to date (85, 86).

##### Criterion 4

This criterion used a cocktail of ESAT6/CFP10 antigens (77) produced by the Italian National Reference Centre for Bovine Tuberculosis at Istituto Zooprofilattico Sperimentale della Lombardia ed Emilia Romagna for the *in vitro* stimulation of heparinized blood. To define the positive sample the recommended cut-off was a net difference of ESAT6/CFP10–PBS ≥ 0.1 OD.

#### Post-mortem Diagnostic Tests

All the buffaloes found to be positive on SITT and had at least one positive result among the two confirmation tests (SICTT and IFN-γ test) were slaughtered and underwent post-mortem examination by official veterinarians to detect the presence of typical bTB lesions. Tissue samples (tonsils, retropharyngeal, mandibular, tracheobronchial, mediastinal, mesenteric, hepatic, sub-iliac, supramammary, popliteal, prescapular lymph nodes, lung, liver, and spleen) were collected for culture of *M. bovis*. The samples were transported to the laboratory and processed within 24 h or frozen at −80°C and then processed according to OIE manual protocols (17). Tissue and organs underwent culture examination and a part of the sample was subjected to direct detection of *Mycobacterium tuberculosis* complex (MTC) (87). In case of isolation of Mycobacteria, molecular, and bacteriological identification was performed as described by Boniotti et al. (87).

### Statistical Analyses

The accuracy of the four IFN-γ assays interpretative criteria was evaluated on OD obtained from a total of 947 buffaloes, of which 489 buffaloes, from bTB outbreaks, tested positive at post-mortem examination and 458 bTB free buffaloes belonging to OTF herds.

The following indices were used to estimate the accuracy: Se, Sp, proportion of false positives, proportion of false negatives, area under the curve (AUC), and Youden index.

Binomial distribution was used to calculate the exact confidence limit of each proportion.

To compare the four IFN-γ assays interpretative criteria for the ROC curve analysis was performed.

Difference between the AUC for each criterion and AUC confidence limit were calculated using the package pRoc of R (88).

For the purpose of the study, Sp was defined as the proportion of samples with negative results from the expected true negative animals, while Se was defined as the proportion of samples positive results from the expected true positive animals.

Regarding criterion 2 and criterion 3, which also give indeterminate results (IN/ND), we have calculated the overall test yield which describes the probability of obtaining a positive or negative result without taking into consideration false positives or false negatives (89). Hence, we calculated the overall test yield (OTY), the negative yield (YD–), and the positive yield (YD+) as described by Simel et al. (89). The YD+ was defined as the probability of a positive result when the expected true positive animals were tested, while the YD– was the probability of a negative test result when the expected true negative animals were tested.

The agreement between the four IFN-γ assays criteria and the expected results (negative for animals belonging to OTF farm and positive at post-mortem test) was estimated on 947 animals, using Cohen's Kappa index and the McNemar-test by proc freq agree of commercial software SAS® version 4.1. A kappa value of 1 indicates perfect agreement and a value of 0 indicates no agreement beyond chance; according with McHugh (90), for the interpretation of the Kappa values, we considered a satisfied level of agreement as a kappa value > 0.9. Kappa Value between criteria were shown by heat map.

Additionally, to assess the Se of all ante-mortem diagnostic tests used in this study, in the subpopulation of *M. bovis*-infected buffaloes, which were SITT and post-mortem positive, we performed a comparison among IFN-γ test, SICTT, and SITT_42_. Of these, SITT_42_ is the result of the bovine PPD inoculation reaction obtained by SICTT, performed 42 days after the SITT screening.

Hence, we compared the results of the IFN-γ test obtained using four interpretative criteria and the readings of SICTT and the SITT_42_. All tests were performed 42 days after the SITT screening. Se values for each test were compared using the binomial exact test.

The precision of the four IFN-γ assays criteria was also estimated in terms of reproducibility and repeatability. Reproducibility was calculated for each criterion on 32 plasma samples from 32 buffaloes: 16 positive and 16 negative from two different laboratories (Istituto Zooprofilattico Sperimentale del Mezzogiorno and Istituto Zooprofilattico Sperimentale dell'Umbria e delle Marche). The Kappa index test was used to quantify the degree of agreement between laboratories on the same sample.

Repeatability was calculated on 12 plasma samples from 12 animals, 6 of which were positive, with different OD values, 2 high, 2 medium and 2 low, and 6 negatives were randomly selected in a panel of negative samples, and three replicates were carried out by the same operator under the same test conditions. For the purpose of the study, repeatability was defined as the degree of agreement between different replicates on the same sample by the same operator, and was calculated by means of the Kappa index. Difference between proportions (Se, Sp, accuracy) was assessed by means of a binomial exact test.

## Results

### Accuracy of Four Interpretative Criteria of the IFN-γ Test

The accuracy of IFN-γ assays according to four interpretative criteria was assessed in 947 animals, 489 were expected to be true positive and 458 expected true negative. Tables 2A,B, 3 shows the estimates of the parameters used to calculate the accuracy.

**Table 2A:** Accuracy of IFN-γ assays according to 4 criteria evaluated in 947 animals.

**TEST**	***N***	**TP**	**TN**	**FP**	**%FP**	**FN**	**%FN**	**IN/ND**	**SE% (CI 95%)**	**SP% (CI 95%)**	***Y*%**
Criterion 1	947	463	451	7	1.5	26	5.3		94.7 (92.3–96.5)	98.5 (98.5–96.9)	93.2
Criterion 2	947	468	313	19	5.7	10	2.1	137	97.9 (96.2–99.0)	94.3 (94.3–91.2)	92.2
Criterion 3	947	428	320	8	2.4	7	1.6	184	98.4 (96.7–99.4)	97.6 (97.6–95.3)	96.0
Criterion 4	947	368	445	13	2.8	121	24.7		75.3 (71.2–79.0)	97.2 (97.2–95.2)	72.4

*N, No of animals tested; TP, true positive; TN, true negative; FP, false positive; %FP, % false positive; FN, false negative; %FN, % false negative; IN, inconclusive; ND, not discriminant; SE CI 95%, sensitivity and confidence interval 95%; SP CI 95%, specificity and confidence interval 95%; and Y, Youden's J*.

**Table 2B:** Accuracy of IFN-γ assays according to 4 criteria evaluated in 947 animals.

**TEST**	***N***	**IN/ND**	**Cut-off**	**PPV% (CI 95%)**	**NPV% (CI 95%)**	**AUC (CI 95%)**
Criterion 1	947		0.1	98.50 (97.40–99.60)	94.50 (92.50–96.60)	0.966 (0.954–0.977)^a^
Criterion 2	947	137	0.05	96.10 (94.40–97.80)	96.90 (95.00–98.80)	0.961 (0.947–0.975)^a^
Criterion 3	947	184		98.20 (96.90–99.40)	97.90 (96.30–99.40)	0.98 (0.97–0.99)^a^
Criterion 4	947		0.1	96.60 (94.80–98.40)	78.60 (75.20–82.00)	0.862 (0.841–0.883)^b^

*N, No of animals tested; IN, inconclusive; ND, not discriminant; PPV CI 95%, positive predictive value and confidence interval 95%; NPV CI 95%, negative predicted value and confidence interval 95%; and AUC CI 95%, AUC and confidence interval 95%*.

a, b *Criterion with same letter are not statistically significant*.

**Table 3:** Results of IFN-γ assays according to Criterion 2 and Criterion 3 on 947 buffaloes tested for bovine tuberculosis.

	**Expected results**		**Total**
	**Negative**	**Positive**	
**Criterion 2**			
Negative	313	10	323
Inconclusive	126	11	137
Positive	19	468	487
Total	458	489	947
**Criterion 3**			
Negative	320	7	327
ND	130	54	184
Positive	8	428	436
Total	458	489	947

Regarding the first criterion, 914 animals were correctly classified out of the total 947 with an observed accuracy of 96.52% (CI 95% 95.35–97.68%). The McNemar-test results were not statistically significant (*p*-value > 0.05). Hence, no disagreement was observed with expected results and the kappa index showed a high level of agreement (kappa 0.93, CI 95% 0.91–0.96).

Concerning the second criterion, classified 810 animals, 332 were expected true negative and 478 were expected true positive. One hundred and thirty-seven animals were classified as inconclusive (IN). Of the 137 IN animals, 11 were expected true positive and 126 were expected true negative. Criterion 2 correctly classified 781/810 animals and the observed accuracy was 96.42%, (CI 95% 95.14–97.70%). Finally, taking into account IN results, the values for OTY, YD+, and YD– were 85.53% (CI 95% 83.13–87.71%), 97.75% (CI 95% 96.01–98.87%), and 72.49% (CI 95% 68.15–76.53%), respectively. The McNemar-test results were not statistically significant (*p*-value > 0.05). Hence, no disagreement was observed with expected results and the kappa index showed a high level of agreement (kappa 0.93, CI 95% 0.90–0.96).

For the third criterion, 763 animals were classified, 328 were expected true negative and 435 were expected true positive. The remaining 184 animals were not discriminant (ND). Of the 184 ND animals, 54 were expected true positive and 130 were expected true negative. Related to the classification of animals, criterion 3 correctly classified 748/763 animals, the observed accuracy was 98.00%, (CI 95% 97.01–98.99%). Finally, taking into account ND results, the values for OYT, YD+, and YD– were 80.57% (CI 95% 77.90–83.04%), 88.96% (CI 95% 85.84–91.59%), and 71.62% (CI 95% 67.25–75.70%), respectively. The McNemar-test results were not statistically significant (*p*-value > 0.05). Hence, no disagreement was observed with expected results and the kappa index showed a high level of agreement (kappa 0.96, CI 95% 0.94–0.98).

For the fourth criterion, accuracy was assessed in 947 animals, 458 were expected true negative and 489 expected true positive. Fourth criterion correctly classified 813/947 animals, the observed accuracy was 85.90%, (CI 95% 83.68–88.12%). The McNemar-test resulted statistically significant (*p*-value <0.05). Hence, no agreement was observed with expected results and the kappa index resulted lower than the others 3 criteria (kappa 0.72, CI 95% 0.68–0.76).

**Figure 1:**
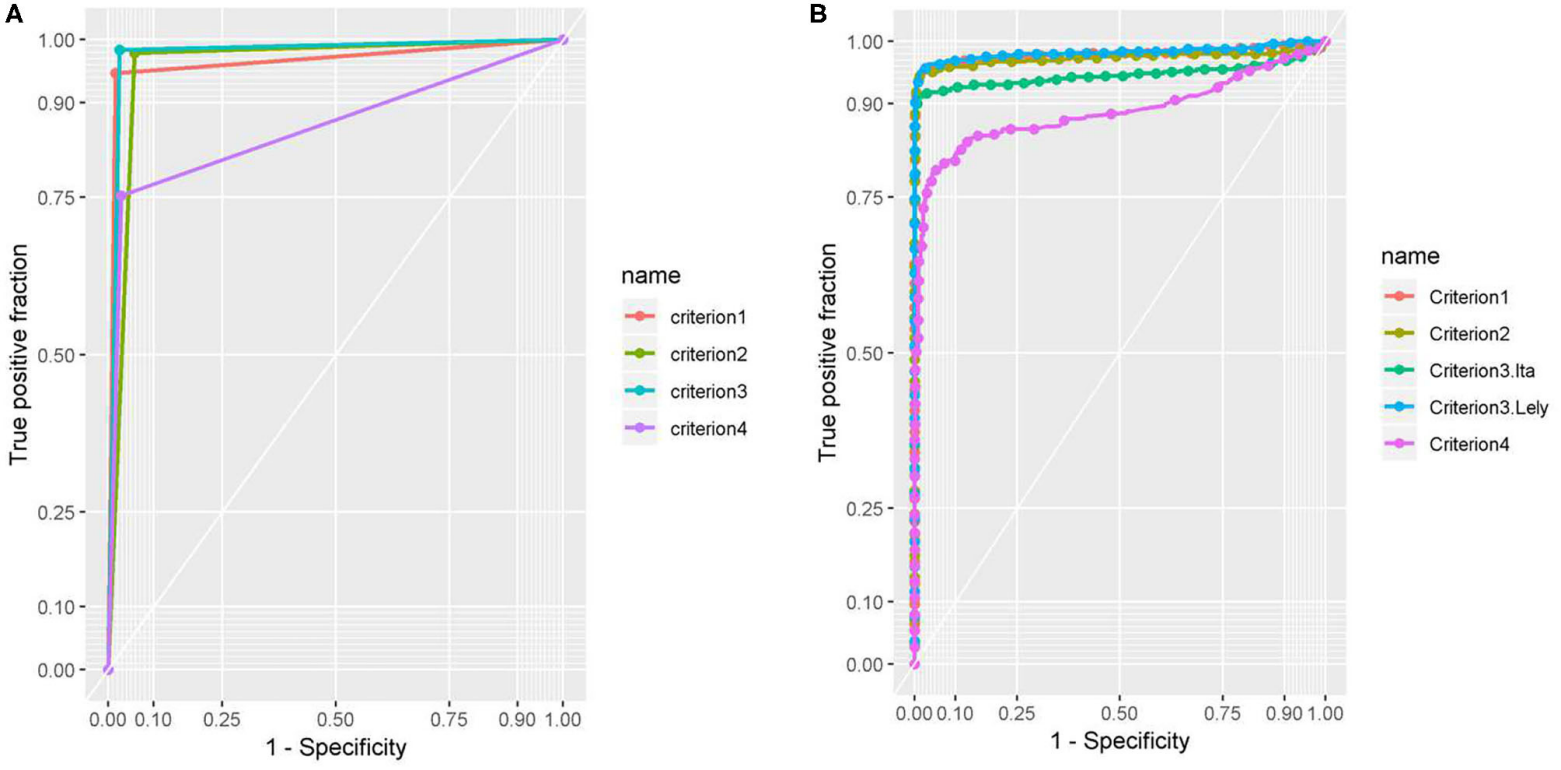
**(A)** The AUC of the four criteria was estimated by binary results for each criteria. **(B)** The AUC of the four criteria was estimated by binary results for each criterion. Test 1 is OD used by criterion 1, test 2 is the OD used by criterion 2, test 3. Lel. is the OD Lelystad PPDs of criterion 3 and test 3. Ita. is the OD Italian PPDs of criterion 3. Test 4 is the OD used by criterion 4.

Results of ROC analysis are shown in Table 4 and Figure 1. No difference resulted between AUC of first criterion, second criterion, and third criterion as DeLong's-test for two correlated ROC curves resulted statistically not significant (*p*-value > 0.05); while AUC of fourth criterion was different with respect to the first, second, and third criterion and the difference was statistically significant (*p*-value < 0.05).

**Table 4:** Accuracy of IFN-γ assays according to 4 criteria evaluated on 718 animals.

**TEST**	***N***	**TP**	**TN**	**FP**	**%FP**	**FN**	**%FN**	**SE% (CI 95%)**	**SP% (CI95%)**	**Youden's J%**
Criterion 1	718	424	281	3	1.1	10	2.3	97.7 (95.8–98.9)	98.9 (96.9–98.9)	96.6
Criterion 2	718	428	276	8	2.8	6	1.4	98.6 (97.0–99.5)	97.2 (94.5–97.2)	95.8
Criterion 3	718	428	276	8	2.8	6	1.4	98.6 (97.0–99.5)	97.2 (94.5–97.2)	95.8
Criterion 4	718	345	280	4	1.4	89	20.5	79.5 (75.4–83.2)	98.6 (96.4–98.6)	78.1

*N, No of animals tested; TP, true positive; TN, true negative; FP, false positive; %FP, % false positive; FN, false negative; %FN, % false negative; SE CI 95%, sensitivity and confidence interval 95%; SP CI 95%, specificity and confidence interval 95%; and Y, Youden's J*.

**Figure 2:**
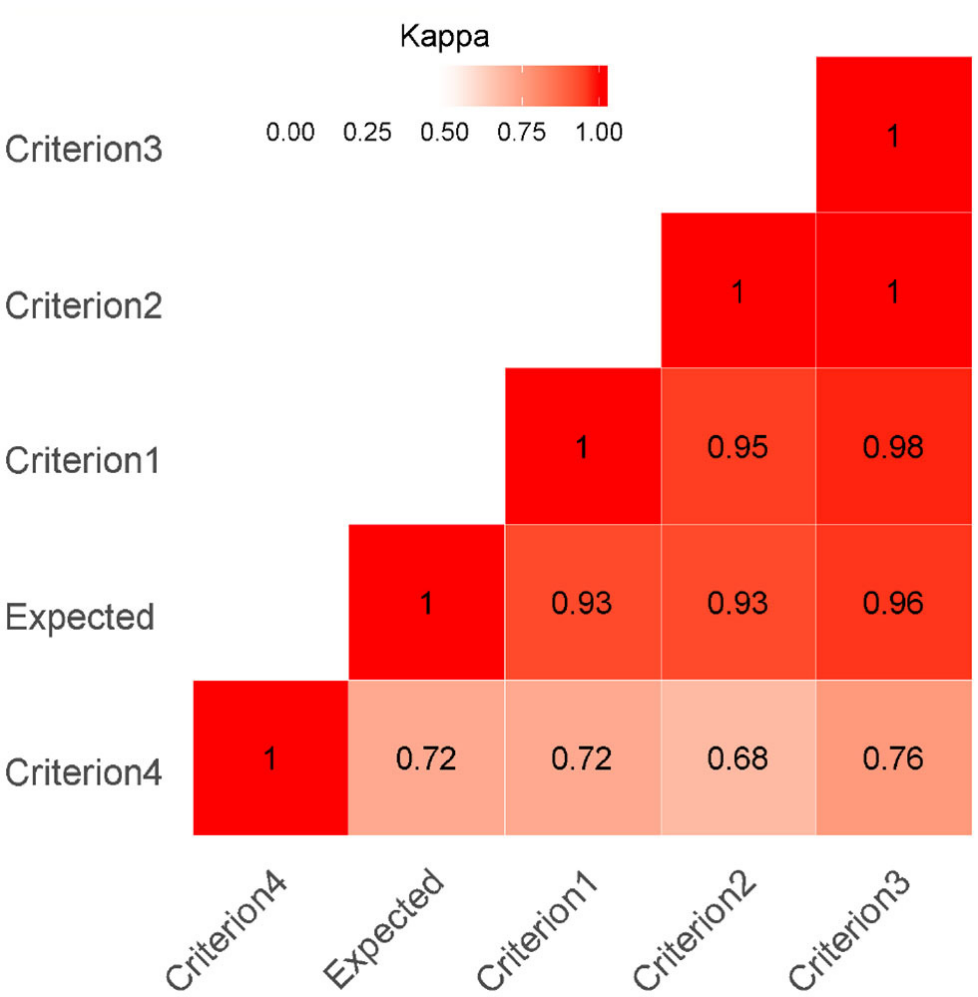
Heat Map Kappa index as a measure of agreement was calculated on 947 buffaloes. A kappa value of 1 indicates perfect agreement and a value of 0 indicates no agreement beyond chance. The red color highlights the agreement (darker reds indicate a stronger agreement of Kappa value between criteria and expected value).

The values of agreement by Kappa between criteria and between the observed and expected results are shown in Figure 2 by heat map.

First, second, and third criterion showed higher level of agreement between them (kappa > 0.95). The Kappa agreement between the observed results of the first three criteria and the expected results was very satisfactory (Kappa > 0.93); hence, the agreement beyond chance was very high. Related to criterion 4, the kappa value ranged from 0.68 (with the second criterion) to 0.76 (with the third criterion), and these values showed an acceptable level of agreement.

The agreement between observed results of the criterion 4 and the expected results was sufficient (Kappa > 0.72).

To better compare the 4 criteria on the same number of animals, the animals that resulted IN in the second criterion and ND in the third criterion were removed from the analysis and the accuracy was evaluated in 718 animals; the results are represented in Table 5.

**Table 5:** Results obtained from 489 *M. bovis*-infected animals, tested 42 days after the SITT screening, by IFN-γ assays, SITT _42_, and SICTT.

**TEST**	***N***	**IN/ND**	**TP**	**FN**	**%FN**	**SE%**	**SE%CI 95%**	
SITT42	489	22	449	18	3.9	96.1	94.0	97.7
SICTT	489	140	308	41	11.7	88.3	84.4	91.4
Criterion 1	489	0	463	26	5.3	94.7	92.3	96.5
Criterion 2	489	11	468	10	2.1	97.9	96.2	99.0
Criterion 3	489	54	428	7	1.6	98.4	96.7	99.4
Criterion 4	489	0	368	121	24.7	75.3	71.2	79.0

*N, No of animals tested; IN, inconclusive; ND, not discriminant; TP, true positive; FN, false negative; %FN, % false negative; and SE CI 95%, sensitivity and confidence interval of 95%*.

#### Criterion 1

Criterion 1 correctly classified 705 out of 718 animals (98.19%, CI 95% 96.92–99.03%). The McNemar-test results were not statistically significant (*p*-value > 0.05). Hence, no disagreement was observed with expected results and the kappa index showed a high level of agreement (kappa 0.96, CI 95% 0.94–0.98).

#### Criterion 2 and Criterion 3

Criterion 2 and criterion 3 showed the same results and correctly classified 704 out of 718 animals (98.05%, CI 95% 96.75–98.93%). The McNemar-test results were not statistically significant (*p*-value > 0.05). Hence, no disagreement was observed with expected results and the kappa index showed a high level of agreement (kappa 0.96, CI 95% 0.94–0.98).

#### Criterion 4

Criterion 4 correctly classified 625 out of 718 animals (87%, CI 95% 84.37–89.42%). The McNemar-test results were statistically significant (*p*-value < 0.05). Hence, no agreement was observed with expected results and the kappa index resulted lower than the other 3 criteria (kappa 0.74, CI 95% 0.69–0.79).

In these animals, the performance of first, second, and third criterion, resulted the same, as the difference between criterion 1, 2, and 3 resulted statistically not significant binomial exact test *p*-value (>0.05).

At the time of initial entry in the herds (screening test), all 489 buffaloes resulted SITT positive. All 489 buffaloes were retested, according to the Campania decree, after 42 days using SICTT and the IFN-γ test.

After 42 days, considering only the bovine PPD inoculation reaction in SICTT (SITT_42_), positive animals decreased from 489 to 449 (449/489, 91.8%), as 18 animals resulted negative (18/489, 3.7%) and 22 animals (22/489, 4.5%) resulted inconclusive. As shown in Table 5, the Se of SITT_42_ was 96.1% (CI 95%, 94.0–97.7%) and that of SICTT was 88.3% (CI 95%, 84.4–91.4%), with 41 false negative and 140 inconclusive results.

The Se of SITT_42_ (96.1%) resulted higher than that by criterion 1 (94.7%), but the difference was not statistically significant (exact binomial test one-sided *p*-value = 0.08). The Se of SITT_42_ (96.1%) resulted lower than that by criterion 2 (97.9%), and the difference was statistically significant (exact binomial test one-sided *p*-value < 0.02). The Se of SITT_42_ (96.1%) resulted lower than that by criterion 3 (98.4%), and the difference was statistically significant (exact binomial test one-sided *p*-value < 0.005). The Se of SITT_42_ (96.1%) resulted higher than that by criterion 4 (75.3%), and the difference was statistically significant (exact binomial test one-sided *p*-value < 0.0001).

### Repeatability and Reproducibility

For interpretative criterion 1, Kappa was 0.81 (CI 95% 0.61–1.00), indicating an almost perfect agreement between the laboratories; 3 discrepancies were observed in 32 samples. For interpretative criterion 2, Kappa was 0.93 (CI 95% 0.83–1.00), indicating an almost perfect agreement between the laboratories, only 1 disagreement was observed in 32 samples. For interpretative criterion 3, the value of K was 1.00 (CI 95% 0.99–1.00), indicating a perfect agreement between the laboratories. For criterion 4, Kappa was 0.87 (CI 95% 0.70–1.00), indicating an almost perfect agreement between the laboratories. According to the interpretation of Landis and Koch, Kappa values between 0.81 and 1 indicate an almost perfect degree of agreement; the reading of the results of IFN-γ therefore proved reproducible for each of the 4 interpretative criteria.

The Fleiss K index was calculated in 12 samples tested by the same operator at 3 different time points. The Fleiss *K*-value was 1.00 (CI 95% 0.67–1.00), indicating an almost perfect match.

### Assessment of the Performance of SITT and IFN-γ on an OTF Farm Where MAP Circulated

In total, SITT was executed in 103 buffaloes, and 102 heparinized blood samples were stimulated; as 8 samples were removed from analysis owing to lack of reaction of lymphocytes against the mitogen, comparison was made in 95 animals. The results of this evaluation are shown in Table 6. On SITT, 43 animals tested negative, while 33 were proved as positive and 19 as inconclusive.

**Table 6:** Number of animals assessed (N), True negative (TN), false positive (FP), number of IN or ND, specificity (SP) and CI 95% SP for SITT and 4 IFN-y test criteria estimated on a sample of 95 OTF animals from a buffalo herd with MAP infection.

**TEST**	***N***	**TN**	**FP**	**%FP**	**IN/ND**	**SP%**	**CI 95% SP%**	
SITT	95	43	52	54.7%		45.3	35.0	55.7
Criterion 1	95	94	1	1.1%		98.9	94.3	99.9
Criterion 2	95	81	2	2.4%	12	97.6	91.6	99.7
Criterion 3	95	58	2	3.3%	35	96.7	88.5	99.6
Criterion 4	95	95	0	0.0%		100	96.2	100

The Sp of SITT in this sample was 45.3% (CI 95% 35.0–55.7%), while INF-γ showed higher Sp according to all of the 4 interpretative criteria. The criterion 1 correctly classified 94 of the 95 animals. Therefore, Sp was 98.9% (CI 95% 93.4–99.9). Criterion 2 correctly classified 81 of the 95 samples; 2 animals were proved as positive and 12 IN. The Sp was 97.6% (CI 95% 91.6–99.7). Criterion 3 correctly classified 58 out of the 60 animals, as 2 animals proved positive and 35 were ND. The Sp was 96.7% (CI 95% 88.4–99.5). Finally, criterion 4 correctly classified all samples; Sp of 100% (CI 95% 96.2–100). Although the Sp varied in IFN-γ evaluation, there were no statistically significant differences observed among criteria 1, 3, and 4 (binomial exact test *p*-value > 0.05). Moreover, between criterion 2 and 3, the confidence intervals overlapped and therefore no significant difference was observed between the Sp-values of the tests (binomial exact test *p*-value > 0.05).

A statistically significant difference was, however, observed between IFN-γ (all criteria) and SITT (binomial exact test *p*-value < 0.05). Agreement between SITT and IFN-γ also proved to be scant, as the Kappa value between SITT and IFN-γ ranged between *k* = 0 for criterion 4, *k* = 0.021 for criterion 1, *k* = 0.033 for criterion 3 and *k* = 0.04 for criterion 2.

## Discussion

In some areas of Italy, the water buffalo is a great economic resource, as mozzarella cheese is prepared from its milk. Apart from the economic standpoint of production losses, the presence of bTB in this species is of great concern for human health. Therefore, it is necessary to implement efficient control measures to support the eradication of the disease in this species. The improvement and assessment of diagnostic techniques are the key steps, especially under field conditions, in the detection of all the infected animals present in a herd, in order to eradicate bTB.

The use of a diagnostic test as well as the set of its cut-off value to define the infectious status of an animal, requires a trade-off between the risk of keeping positive animals in the herds (Sensitivity) and the risk of slaughtering negative animals (Specificity), based on epidemiological context and local legislation (15, 29, 53).

For this reason, it is essential to provide legislators and official veterinarians with a flexible tool that, depending on the epidemiological context, allows them to decide whether to favor Se or Sp. With this aim, it was decided to combine the traditional intradermal skin tests (SITT and SICTT) with the IFN-γ test to increase the accuracy of bTB diagnostic investigation both at herd and individual level.

This study is the first to describe the evaluation of the IFN-γ assay in the diagnosis of bTB in water buffalo, comparing 4 different interpretative criteria. In particular we evaluated the accuracy of 4 interpretative criteria for the IFN-γ test in buffaloes from *M. bovis*-infected herds and OTF ones, under field conditions.

The 4 criteria chosen to evaluate the performance of the IFN-γ test have been validated in cattle and are currently used in the diagnosis of bTB in this species, in particular the first criterion is the one suggested by the manufacturer; the second is used at the Italian National Reference Center for bTB in Italy but has also been used in Spain (29, 52, 79, 80); the third is currently used at Istituto Zooprofilattico Sperimentale del Piemonte, Liguria e Valle d'Aosta laboratory and was used to eradicate bTB in Piedmont in the years from 2004 to 2016, when the region acquired the European OTF status according to Decision 2016/168 (85, 86); the fourth uses a cocktail of ESAT6/CFP10 antigens (77) for the *in vitro* stimulation of heparinized blood, it is widely used in the IFN-γ assay to improve Sp (16, 30, 62, 63).

To assess the Se of the four criteria, in bTB infected herds in SITT positive buffalo, we defined as bTB positive animals those in which were detected bTB typical lesions and/or *M. bovis* was detected by culture or PCR, introduced only recently (87).

Although the population of subjects included in the Se assessment had been selected from animals that had already tested positive in the screening SITT test, and this may have overestimated the Se of the IFN-γ test, it was still possible to fulfill the goal of this study, the comparison between the 4 criteria.

The Se of culture in the case of *M. bovis* was very low, ranging from 58.0 to 80%, on the basis of culture media and the decontamination procedure used (67, 68). This Se limitation influences the assessment of the IFN-γ test performance leading to a misclassification of data (91). Often for the IFN-γ test, the reported ranges were from 73.0 to 100% for Se and from 85.0 to 99.6% for Sp (15). This variability depends on differences in cattle populations and cut-off values adopted in the interpretation of results as well as the gold standard used for classification of bTB infection status (92). Hence, we decided to take into account other diagnostic tests to define the bTB positive buffalo.

To assess the Sp, a negative animal was defined as a buffalo from an OTF herd (at least in the last 6 years), tested negative to the SITT at the last official control.

Therefore, we defined the accuracy of the 4 interpretative criteria of the IFN-γ test in 489 expected true positive and 458 expected true negative animals, and we compered the results of the four criteria with the expected results.

The IFN-γ test parameter estimates had high Se and Sp according to all interpretative criteria (Tables 2A,B). In particular, the four interpretative criteria of the IFN-γ test showed high levels of accuracy, with Se levels ranging from 75.3% for the fourth criterion (CI 95% 71.2–79.0%) and Se 98.4% for the third criterion (CI 95% 96.7–99.4%); Sp levels were between 94.3% for the second criterion (CI 95% 91.2–96.50%) and Sp 98.5% for the first criterion (CI 95% 96.9–99.4%).

Further, we evaluated the accuracy of the criteria with the AUC analysis, the Youden index, the agreement between the observed, and the expected results from McNemar-test and the Kappa index.

For all indicators the first three criteria showed high level of accuracy, while the fourth criterion lacked Se, and showed lower level of accuracy.

Comparing the results among the criteria, the AUC of the first three criteria were very similar, while AUC of the fourth criterion resulted in a statistically significant difference (*p*-value < 0.05) with respect to the other criteria; same results were also observed by the agreement between the first three criteria (Kappa > 0.95).

Regarding the agreement between the observed results of the first three criteria and the expected results was very satisfactory (Kappa > 0.93) as high levels of Se and Sp were gained. Moreover, the agreement between observed results of the criterion 4 and the expected results was sufficient (Kappa > 0.72), and this was especially due to the lower values of Se (75.3%, CI 95% 71.2–79.0%).

Although the differences between the first three criteria were not statistically significant (*p*-value > 0.05), it is possible to observe that there are criteria capable to reveal a larger number of infected animals and criteria that, being more specific, leave many infected animals in herd (Table 2A).

In particular in criteria 1 and 4, respectively, the 2.75% (26/947) and 12.78% (121/947) of positive animals are not correctly identified. Therefore, these criteria would be more appropriate for situations with low bTB prevalence or in the final stages of a disease eradication plan.

On the contrary the second and the third criteria, which provide a set of inconclusive results, leave a lower number of positive animals in the herd [1.05% (10/947) and 0.74% (7/947), respectively]. Therefore, these criteria should be used in high bTB prevalence context either in herds or territories.

The same performance was observed in the data-set where the inconclusive results had been removed, and the assessment of the accuracy was performed in 718 animals (Table 3).

In fact, the second and third criteria introduce the possibility of animals without an outcome, the second criterion gives 14.5% (137/947) of IN results and the third criterion 19.4% (184/947) ND results. In general, these animals are more difficult to define. When evaluating all the criteria these “difficult animals” were removed and the criterion 2 and 3 had shown better values of Se (98.6% CI 95% 97.0–99.5%) in contrast to the criterion 1 (97.7% CI 95% 95.8–98.9%) and criterion 4 (79.5% CI 95% 75.4–83.2%).

However, with the second criterion, among 137 IN animals, only 11 were expected true positive and the other 126 were expected true negative. For the criterion 3, which is based on the relationship between two couples of PPDB and PPDA, among the 184 ND animals, 54 were expected true positive and 130 were expected true negative, because this criterion is the most conservative one. In fact, this criterion provides different steps and controls to define the correct sample and result; in terms of accuracy, it therefore achieves better performance (fewer false positives), but leaves more subjects without an outcome. Similar to other reports (16, 32) a maximum threshold of the basal value (PBS ≤ 0.150 OD) has been introduced in this criterion, an additional quality control, to make the test more “conservative” and therefore not consider suitable animals with high basal values (PBS) due to pre-existing pathologies. For these animals the official veterinarians have to repeat the blood sampling at least after 42 days from the last SITT or SICTT, this leads to longer recovery times but more accurate outcomes.

In addition, our study also suggests that using the ESAT6/CFP10 cocktail (fourth criterion), in addition to PPDs, minimizes the possibility of obtaining a false-positive result. It could therefore be a useful tool for diagnosing bTB in herds or territories in which the prevalence of bTB is low.

The four criteria also showed high levels of precision as the reproducibility and repeatability values were very satisfactory, and the tests were carried out in accredited public laboratories that have been performing the IFN-γ test for several years.

Since a major limitation to the interpretation of the *in vivo* and *in vitro* bTB assays is the cross-reactivity with responses induced by exposure to NTM, including MAP, we wanted to assess the Sp of the 4 criteria of the IFN-γ test in a particular but frequent situation a buffalo herd negative to bTB (OTF) but PTB infected for several years. In buffalo, as in cattle, infection/exposure to NTM can interfere with bTB diagnosis, because the composition of PPDs includes several antigens that can cross-react with environmental mycobacteria and this may lead to false positive reactions (93). MAP, the causative agent of PTB, is one of the most important NTM causes of false positive reactions to PPD in cattle and buffalo (11, 12, 69–75).

The data analyzed in one OTF herd MAP-infected, showed that the Sp of SITT was 45.3% (CI 95% 35.0–55.7%) lower than the values reported in cattle in the literature between 75.5 and 99.0% (15).

The lack of concordance between the Sp of the IFN-γ test (96.7% CI 95% 88.5% 89.6–100% CI95% 96.20–100%) and the Sp of SITT (45.3% CI 95% 35.0–55.7%) in the OTF herds with MAP infection was due to the use of the avian PPDs in the IFN-γ test, that were able to correctly identify MAP-infected animals and therefore classify them as *M. bovis-*negative (15, 16, 69, 94).

In summary, our data indicate that, in buffalo, the IFN-γ assay is an excellent test and shows good accuracy which ranged from 96.42% (CI95% 95.14-97.70%) to 98.00% (CI95% 97.01-98.99%) for the three best criteria.

Since in cattle, the diagnostic Se of bTB positive animals improves when SITT is used in combination with the IFN-γ test (38, 82), we can assume that the same can also occur in buffalo. This consideration is also supported by the performance of the IFN-γ test obtained in our investigation in buffalo.

In the present study, the Se of the IFN-γ test in buffalo, which ranged from 94.70% (CI95% 92.30-96.50%) to 98.04% (CI95% 96.70-99.40%) for the best three criteria, was comparable to that indicated in cattle, with a Se estimated median of 87.6% with a range between 73 and 100% (15, 30).

With regard to Sp, our values in the buffalo ranged between 97.20% (CI95% 95.20-98.50%) to 98.50% (CI95% 96.90-99.40%) for the best three criteria, and we have achieved the best performance reported in cattle, with a Sp median value of 96.6% (range: 85.0–99.6%) (15, 30).

Moreover, taking into account that our Se values could be overestimated due to the obligate selection of animals already tested positive in the SIT screening test, our data in the *B. bubalis* displayed higher Se and Sp levels than those reported in *S. caffer* by Michael et al. (35) (92.1% Se and 68.3% Sp) and similar to the relative Se values of 100% reported by van der Heijden et al. (37).

In order to implement the results of our IFN-γ test performance evaluations, we are looking for suspected or confirmed infected buffalo herds where we can perform simultaneously IFN-γ and SITT tests on all animals of the herd.

In cattle the IFN-γ assay is incorporated into a lot of national bTB eradication programs (15, 29, 33). In particular, in epidemiological context in which the prevalence of bTB is high, but also in the extinction phase of a bTB outbreak, SITT or SICTT could be used together with the IFN-γ test. In such situations, in order to obtain higher Se, it would be useful to consider the “tests in parallel,” and to classify as positive those animals that react to at least one test (37, 38, 40). By contrast, in areas where the prevalence of bTB is low, or in bTB-free herds, it would still be appropriate to use SITT or SICTT together with the IFN-γ test, it would be preferable to consider the “tests in series” and to classify as positive only animals that react to both tests, thereby improving Sp (17).

The differences in the SITT readings between the first test and the second one performed after 42 days (SITT_42_), explain why the usefulness of SITT in the diagnosis of bTB in buffaloes is still debated (10, 38, 95). In fact, among the buffaloes that resulted positive to the SITT screening test, 18 animals resulted negative and 22 inconclusive to SITT_42_.

These findings confirm how the SITT readings are difficult to interpret, in buffaloes, due to the tissue structure, varying thickness, and black color of the skin. Furthermore, SITT is a subjective test, because the interpretation of the reaction to PPD inoculation may vary between operators. Instead, the IFN-γ test is an objective laboratory test, which provides readings with instruments that prevent a subjective evaluation of the results (57, 78).

Several studies (18, 26, 96, 97) have shown that SICTT-negative/IFN-γ-positive animals have a 2- to 10-fold higher risk of being *M. bovis-*infected. Therefore, given the high level of risk associated with keeping SICTT-negative/IFN-γ-positive animals in an infected herd, the rapid removal of these animals appears to be the most effective measure. This reduces the potential for transmission within the herd and the future risk of recurrence of bTB infection, and to avoid a longer period of restriction or to avoid causing restriction in another herd as a result of movements.

A similar pattern of results was also obtained in our study; among the 489 buffaloes that proved to be infected with *M. bovis*, the SICTT showed low Se (88.3%; CI 95%, 84.4–91.4%) due to the highest number of inconclusive results (140/489) and 41 false-negative results (Table 4).

The four criteria of the IFN-γ test, among the 140 inconclusive SICTT results, detected 121 bTB positive animals (mean of the results using the four criteria) and among the 41 false-negative results, identified a mean of 36 bTB positive buffaloes.

The IFN-γ test has been proved as an objective method, as it utilizes a standard procedure and is not affected by the subjectivity of the operator, in contrast to SITT, which could be influenced by several factors that can interfere with Sp and Se (15, 30). Moreover, the IFN-γ test has a short execution time and can be repeated without time constraints. Unlike SITT, it does not interfere with the immune profile of the animal. In addition, it is not influenced by treatments with immunosuppressive drugs and is not affected, or at least is much less affected, by infection with mycobacteria other than *M. bovis*. Furthermore, its different interpretative criteria and antigens can be adopted according to the objective to be pursued and the epidemiological context (39). Our results in buffalo indicate that an IFN-γ-positive animal, especially if the test is applied in a bTB-infected herd, has a very high probability of really being infected (Table 2B).

Finally, countries that gained bTB eradication in cattle, including Australia, focused their attention on the herd rather than on the individual animal; these countries had considered SITT as primary screening test for bTB in herds because of its low accuracy (98), while at individual level, to maximize the detection of infected animals, they used the IFN-γ assay (17, 68).

## Conclusions

In summary, our study provides new data on the Se and Sp of the IFN-γ test comparing four interpretative criteria for bTB diagnosis in water buffalo under field conditions.

Our results showed that the IFN-γ test in the buffalo species could reach high Se and Sp values, and that the level of Se and Sp could be chosen according to the interpretative criterion and the antigens used, depending on the health status of the herd and the epidemiological context of the territory.

In addition, the 4 interpretive criteria, in OTF herds with PTB, proved to be particularly useful in drastically reducing false positivity reaction for *M. bovis* compared to SITT.

Based on our results, in order to improve bTB diagnostic Se in buffalo herds, IFN-γ assays could be used in parallel with the SITT to identify the largest number of infected buffaloes in bTB outbreaks. Meanwhile, in order to improve bTB diagnostic Sp, IFN-γ assays could be used in series with SITT to limit false positive results in buffalo herds that are officially bTB-free.

Starting from the reported experience in cattle and the data of our study, in territories where bTB is still present, such as the Campania region, the use of the IFN-γ assay can support successfully the bTB eradication programme in buffalo.

## Data Availability Statement

The raw data supporting the conclusions of this article will be made available by the authors, without undue reservation.

## Ethics Statement

The SITT and SICTT and collection of blood samples on which the IFN-tests were carried out was conducted as part of The National and Regional Buffalo TB Eradication Programme, in compliance with the EU trade (14) Council Directive 64/432/EEC, which governs the nature and frequency of testing. Written informed consent was obtained from the owners for the participation of their animals in this study.

## Author Contributions

AM, ED, MP, MB, PM, and NV carried out the conceptualization of the study and performed the supervision. AM, PM, AD, IA, LS, and AC developed the methodology. NV and LC dealt with the software. AM, AD, NV, IA, FG, LS, and PM carried out the validation. NV, LC, AM, PM, MB, and MP performed the formal analysis. AM, AD, IA, LS, AC, and FG performed the investigation. AM, ED, and PM addressed the resources. NV, LC, AM, and LS performed the data care. AM, NV, PM, IA, MB, and MP prepared the original drafts for writing. AM, NV, PM, MB, MP, and ED carried out the writing—review and editing. All authors contributed to the article and approved the submitted version.

## Conflict of Interest

The authors declare that the research was conducted in the absence of any commercial or financial relationships that could be construed as a potential conflict of interest.

## Footnotes

^1^Campania Region Executive Decree 236 of 14 October 2016. Procedures on the application of Community, national, regional and national legislation for the eradication of tuberculosis in cattle and buffaloes—amendment annexed to DD 226 of 3 October 2016.

^2^Campania Region Executive Decree 226 of 3 October 2016. Procedures on the application of Community, national, regional legislation for the eradication of tuberculosis in cattle and buffaloes.
